# Computational investigation of the time-dependent contact behaviour of the human tibiofemoral joint under body weight

**DOI:** 10.1177/0954411914559737

**Published:** 2014-11

**Authors:** Qingen Meng, Zhongmin Jin, Ruth Wilcox, John Fisher

**Affiliations:** 1 Institute of Medical and Biological Engineering, School of Mechanical Engineering, University of Leeds, Leeds, UK; 2 School of Mechanical Engineering, Xi’an Jiaotong University, Xi’an, China

**Keywords:** Knee biomechanics, cartilage, meniscus, meniscectomy, finite element modelling

## Abstract

The knee joint is one of the most common sites for osteoarthritis, the onset and progression of which are believed to relate to the mechanical environment of cartilage. To understand this environment, it is necessary to take into account the complex biphasic contact interactions of the cartilage and menisci. In this study, the time-dependent contact behaviour of an intact and a meniscectomized human tibiofemoral joint was characterized under body weight using a computational model. Good agreement in the contact area and femoral displacement under static loads were found between model predictions of this study and published experimental measurements. The time-dependent results indicated that as loading time progressed, the contact area and femoral vertical displacement of both intact and meniscectomized joints increased. More load was transferred to the cartilage–cartilage interface over time. However, the portions of load borne by the lateral and medial compartments did not greatly vary with time. Additionally, during the whole simulation period, the maximum compressive stress in the meniscectomized joint was higher than that in the intact joint. The fluid pressure in the intact and meniscectomized joints remained remarkably high at the condyle centres, but the fluid pressure at the cartilage–meniscus interface decreased faster than that at the condyle centres as loading time progressed. The above findings provide further insights into the mechanical environment of the cartilage and meniscus within the human knee joint.

## Introduction

The knee is one of the most complex articulating joints of the human body. It supports the body and facilitates locomotion for daily activities. It is also a common site for osteoarthritis (OA),^1,2^ which is one of the leading causes of joint pain and disability.^3–5^ Although the aetiology of OA is not fully understood, the onset and progression are generally believed to be related to the mechanical environment within the joint.^6^ The cartilage and meniscus tissues are biphasic, and the fluid phase plays a major role in load support. The fluid pressure also increases the effective stiffness of the cartilage, a reduction of which is clinically identified as an early sign of cartilage degeneration.^7,8^ It is, therefore, important that this biphasic behaviour is taken into account when investigating the progression of OA or when examining the effects of clinical interventions.

Computational models, especially those using finite element (FE) methods, have been developed extensively to study the mechanics of the tibiofemoral joint because they can provide information that would be difficult or impossible to obtain from experimental and clinical studies.^9^ However, there are a number of challenges in using such computational methods.^10^ First, there is a need to represent the cartilage and meniscus as biphasic materials as discussed above. In addition, the collagen fibres within the solid phase provide tensile stiffness, which significantly improves the fluid pressurization of these tissues by restricting the lateral deformation under compressive loading.^11,12^ The differing tension–compression behaviour of the solid phase should also be taken into account, for example, by using a fibril-reinforced model.^10,13,14^ Second, in order to satisfy the balance laws for mass, momentum and energy in modelling the cartilage and meniscus mechanical behaviour, the fluid pressure must be continuous on the interfaces where the cartilage and meniscus components come into contact.^15^ Outside the contact area where the cartilage and meniscus interact with the surrounding fluid, a free-draining boundary condition should be enforced to satisfy the above balance laws.^15–18^ Moreover, the regions that require these different boundary conditions move as the contact area changes. In some commercial software packages, user-defined subroutines are required to implement these contact boundary conditions,^17–19^ and it has been shown that the model solutions differ considerably if the differing contact boundary conditions within and outside the contact area are not included.^19^ Third, the geometries of the components of the tibiofemoral joint are not uniform and regular. Six separate contact pairs (femoral cartilage–meniscus, meniscus–tibial cartilage and femoral cartilage–tibial cartilage on both the lateral and medial compartments) are formed between the articular surfaces of the non-uniform geometries. These contact pairs are not easy to solve even if elastic materials are used for the cartilage and meniscus. In addition, under physiological loading such as body weight (BW), the finite strain (large deformation) theory should be applied to accommodate the large deformation and sliding of the soft tissues.^20^

Previous studies have had to make a number of assumptions to simplify their knee models sufficiently to enable them to be solved. For example, some studies have assumed that the cartilage and meniscus act as elastic materials.^21–23^ Such a simplification is only valid for an instantaneous response where there is no time for the fluid to flow at the instant of loading or at equilibrium when the fluid flow ceases. If the time-dependent response of the joint is sought, this assumption is no longer satisfactory.^24^ A few studies have considered the cartilage and meniscus as biphasic materials.^20,25,26^ However, these studies were limited to low levels of loading values, and the realistic fluid flow contact boundary conditions were not considered.^20^ Another approach has been to model only the cartilage as biphasic with the menisci as a transversely isotropic linear elastic material.^27,28^ These studies also did not specify the free fluid flow boundary condition out of the contact area for the six contact pairs.

Since the articular cartilage and meniscus are both biphasic materials, they manifest time-dependent behaviour even under constant load or displacement. Investigating such behaviour is a widely used approach to characterize the mechanical properties^29,30^ of these tissues. At the whole joint scale, the time-dependent contact behaviour of the tibiofemoral joint under constant BW is physiologically relevant, that is, for two-legged stance over extended periods (occurs during prolonged periods of standing). Such an investigation can provide insight into the mechanical environment of the whole joint and the biomechanical functions of the articular cartilage and meniscus. However, the time-dependent contact behaviour of the tibiofemoral joint under BW with realistic fluid flow contact boundary conditions has yet to be fully investigated.

Therefore, the aim of this study was to develop a FE contact model for the human tibiofemoral joint capable of simulating two-legged stance over long periods with realistic fluid flow contact boundary conditions. The model was used to characterize the time-dependent behaviour of the joint in an intact state and following total meniscectomy.

## Models and methods

All the analyses were undertaken using FEBio (version 1.5.0; Musculoskeletal Research Laboratories, University of Utah, Salt Lake City, UT, USA), which is developed specifically for biomechanical applications and accommodates finite deformation.^31^

### Geometry

The geometry of the investigated human tibiofemoral joint was taken from the Open Knee Project.^32,33^ Magnetic resonance (MR) images of a female donor’s right knee (age 70 years, height 1.68 m and weight 77.1 kg) were collected using a 1.0-T extremity scanner (Orthone; ONI Medical Systems, Inc., Wilmington, MA, USA) with the joint at full extension.^32^ Bone, cartilage and menisci were segmented and reconstructed from the MR images.^32^

### Materials

**Figure 1.**
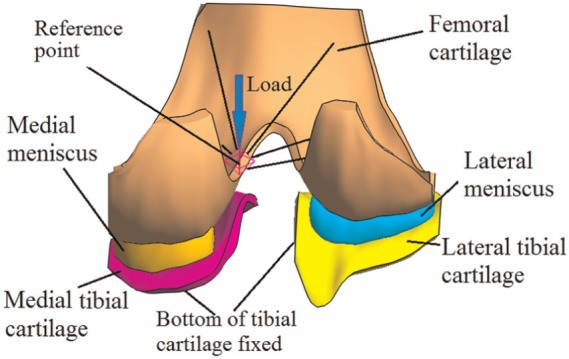
The tibiofemoral model investigated in this study (viewed posteriorly in the direction normal to the coronal plane).

The tibia and femur bones were assumed to be rigid since they are much stiffer than the soft tissues.^21^ In order to simplify the model and solution and reduce computational cost, the ligaments were not considered, but their function to constrain joint motion was taken into account through the loads and boundary conditions applied to the FE model.^27,28,34^ The intact model contained tibial and femoral cartilage and medial and lateral menisci (Figure 1). In the meniscectomy model, a double meniscectomy case was considered. It should be noted that although meniscectomy may be performed due to acute meniscal traumatic injuries,^35^ removing both menisci is an extreme case and rarely performed today.^36^ This extreme and unlikely clinical scenario was chosen only to assess the functional behaviour of the cartilage in isolation, highlight the function of the menisci and demonstrate the sensitivity of the model to pathologic conditions.^37^

The cartilage and menisci were considered as fibril-reinforced biphasic materials. The governing equations for the fibril-reinforced biphasic material used in this study are summarized in Appendix 1. As explained in Appendix 1, the compressive stiffness and Poisson’s ratio of the non-fibrillar matrix, tensile moduli of the collagen fibres and permeability are required to define the material properties of a fibril-reinforced biphasic material. The properties used for the cartilage and menisci are shown in Table 1. They were selected to represent typical values obtained from the available experimental data, taking mid-values or averages where necessary. The equilibrium compressive modulus of the human meniscus can be as small as 0.1 MPa.^38^ However, to avoid the self-contact of the inner surface of the menisci, a higher compressive modulus (1.0 MPa), which is close to the compressive modulus tested at a physiological strain rate,^39^ was assumed in this study.

**Table 1.** Material properties of cartilage and meniscus used in this study.

	Equilibrium compressive modulus (MPa)	Poisson’s ratio	Tensile modulus (MPa)	Permeability (mm^4^/N s)
Femoral cartilage	0.64^a,40^	0.08^a,40^	5.6^b,41^	0.00116^a,40^
Tibial cartilage	0.84^c,42^	0.03^42^	5.6^d,41^	0.00326^42^
Meniscus (only applicable for the intact model)	1.0^39^	0.03^38^	Circumferential: 40.0^43^	0.00100^38^
			Radial: 10.0^e,44^	

a Average values of the medial and lateral condyles.

b Average value from all zones of normal femoral cartilage.

c Also similar to mid-value found by Akizuki et al.^45^

d Due to lack of experimental data, this value was taken from the femoral cartilage.

e Average of posterior, central and anterior regions.

### Contact conditions

In FEBio, the *biphasic* analysis step was used to solve the contact problems. Six biphasic contact pairs were defined for the intact joint: femoral cartilage–meniscus, meniscus–tibial cartilage and femoral cartilage–tibial cartilage on both the lateral and medial sides. For the meniscectomized joint, the two cartilage–cartilage biphasic contact pairs were defined. The *sliding2* implementation, which by default takes large sliding into account, was used for all the contact pairs. For each contact pair, the free-draining boundary condition out of the contact area was satisfied automatically because it is considered in FEBio by default.^16,31^ The penalty method, in which the contact traction is determined by the gap (i.e. the penetration (normal overlapping) distance between the two contacting surfaces) multiplied by the penalty factor, was used to enforce the contact constraints. The auto-penalty was applied for all contact pairs to calculate a suitable initial value for the penalty factor.

### Loading and boundary conditions

The tibiofemoral joint in full extension (i.e. as in two-legged stance) was simulated. The bottom of the tibial cartilage was fully fixed to simulate an ideal bond between the cartilage and the tibial bone. For the femur, the rotation in the flexion–extension direction and the translation in the transverse plane were fixed,^21^ while the vertical (in the superior to inferior direction) translation and internal–external (IE) and varus–valgus (VV) rotations were allowed. The interface between the femoral cartilage and femur was coupled to a reference point, which was used to constrain the femur and apply load. To simulate a physiological loading condition, the reference point was 5 mm medial to the joint centre (Figure 1), which was the midpoint of medial and lateral femoral condyles.^32^ Such a 5-mm offset was consistent with the requirement for wear test of the total knee replacement specified by ISO 14243.^46,47^ Measured with instrumented implants, the contact force of the tibiofemoral joint under two-legged stance is approximately one BW.^48^ Therefore, a vertical load of 800 N, approximately BW, was applied to the reference point. The load was applied over 1 s and kept constant for a further 1200 s. This load was equivalent to a vertical load of 800 N and an adduction (varus) moment of 4 N m applied to the joint centre. The equivalent adduction moment (4 N m) was within the scope of the two-legged stance measured by Kutzner et al.^48^ The effect of the loading position on the contact behaviour of the intact joint was conducted, and the analysis can be found in Appendix 2. Except for the anterior and posterior ends that were fixed in the transverse plane to simulate the constraints of the horn attachments,^25^ the menisci were free to deform in all directions. Free-draining boundary conditions were applied on the peripheral surfaces of the cartilage and menisci.

To assess the validity of the model predictions, in addition to the constant loading over an extended period, three instantaneous loads used in previous experimental studies, 500 N (62.5% BW),^37,49^ 1000 N (125% BW)^37,49,50^ and 1500 N (187.5% BW),^37,49^ were also applied to the model and the outputs compared to data from the literature.

### Mesh

The mesh density adopted was determined after a mesh convergence study. A total of approximately 38,000 hexahedral elements were used for the cartilage and menisci. A further doubling of the element number caused only a 4.3% increase in the peak fluid pressure on the cartilage at the instance when the load was applied. Therefore, the extra computational cost was not justified for this study.

### Outputs

Initially, the predicted femoral vertical displacement and total contact area for the three instantaneous loads were compared with published experimental data. Then, the time-dependent variations in a number of important mechanical parameters related to the contact behaviour of the tibiofemoral joint were characterized. These parameters included the third principal strain in the compartment centres, femoral vertical displacement, contact area, load transmitted by the cartilage–cartilage and cartilage–meniscus interfaces, load distribution between the medial and lateral compartments, maximum compressive stress (the third principal stress) and the fluid pressure of the joints. The ratio of the fluid pressure to the contact pressure (termed ‘fluid support ratio’ in this study) at different locations in the intact and meniscectomized joint was also compared to assess the spatial variation in this parameter over time between the two models.

## Results

The femoral vertical displacements under the three instantaneous loads are presented in Table 2, along with values obtained from the literature. For all three cases investigated, the displacements obtained from the current model were in the range of the experimental tests reported by Kurosawa et al.^49^ and between the values reported by Shrive et al.^52^ and Walker and Erkman.^51^ The comparison of the contact area is shown in Table 3. Under both 500 and 1000 N, the contact area at each compartment and the total contact area predicted by this study agreed very well with the experimental measurements by Fukubayashi and Kurosawa.^37^ For the case of 1000 N, the contact areas at both medial and lateral compartments were also within the range measured in a recent experimental test.^50^

**Table 2.** Comparison of the femoral vertical displacement (mm) under given instantaneous loads between the model predictions in this study and published experiments.

500 N			1000 N			1500 N		
Experiments		This study	Experiments		This study	Experiments		This study
Kurosawa et al.^49^	0.66 ± 0.17	0.79	Kurosawa et al.^49^	0.87 ± 0.17	1.02	Kurosawa et al.^49^	1.04 ± 0.23	1.17
Walker and Erkman^51^	0.42		Walker and Erkman^51^	0.65		Walker and Erkman^51^	0.81	
Shrive et al.^52^	1.0		Shrive et al.^52^	1.28		Shrive et al.^52^	1.56	

The ± values represent a standard deviation; the values of the literature Walker and Erkman^51^ and Shrive et al.^52^ were measured from the curves presented in the articles.

**Table 3.** Comparison of the contact areas (cm^2^) between the model predictions in this study and published experiments under instantaneous loads.

	500 N		1000 N		
	Experiment^37^	This study	Experiment^37^	Experiment^50^	This study
Medial	5.30 ± 1.50	5.63	6.40 ± 1.80	5.95 ± 1.55; 5.61 ± 1.99	6.14
Lateral	4.20 ± 0.60	4.12	5.10 ± 0.70	4.44 ± 1.07; 4.42 ± 1.34	5.21
Total	9.60 ± 1.70	9.78	11.50 ± 2.00	−	11.35

The ± values represent a standard deviation; in the experiment by Morimoto et al.,^50^ two groups of test were performed. Note that all contact areas presented in this article are the sum of cartilage–meniscus and cartilage–cartilage interfaces.

**Figure 2.**
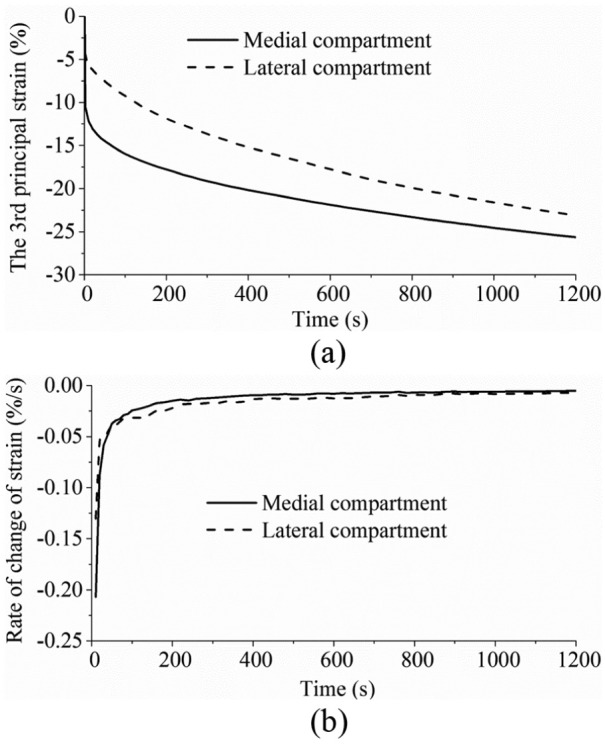
(a) The third principal strain and (b) the rate of change of the third principal strain in the medial and lateral compartment centres over time.

The time-dependent third principal strains in the centre of the medial and lateral compartments are shown in Figure 2(a), and the corresponding rates of change in the third principal strain are shown in Figure 2(b). The third principal strain showed a similar trend to the contact deformation measured by Hosseini et al.:^53^ it increased rapidly when the load was just applied; after a period of time, the rate of change approached zero.

**Figure 3.**
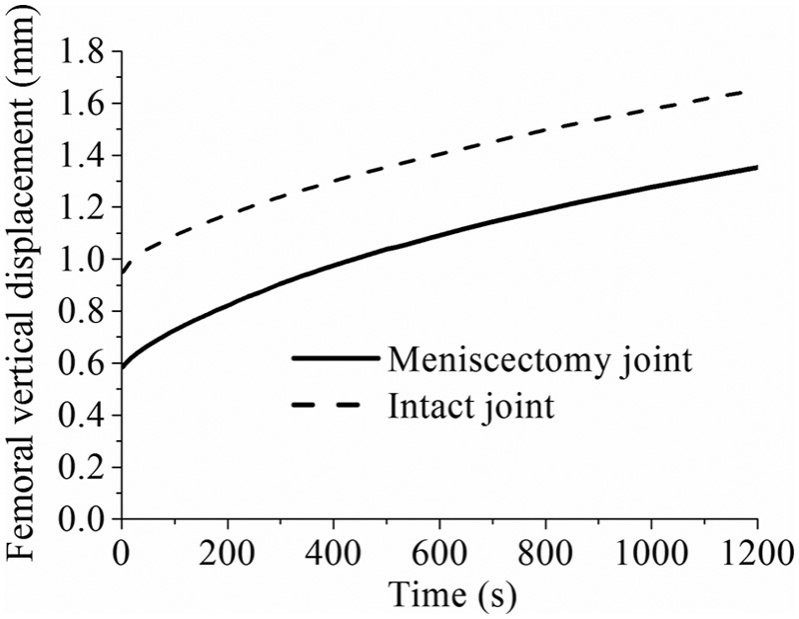
The femoral vertical displacement of the intact and meniscectomy models within 1200 s of creep. The calculation of the displacement of the meniscectomy model started when the femoral and tibial cartilage contacted (the initial gap between the femoral and tibial cartilage caused by removing the menisci was not included).

**Figure 4.**
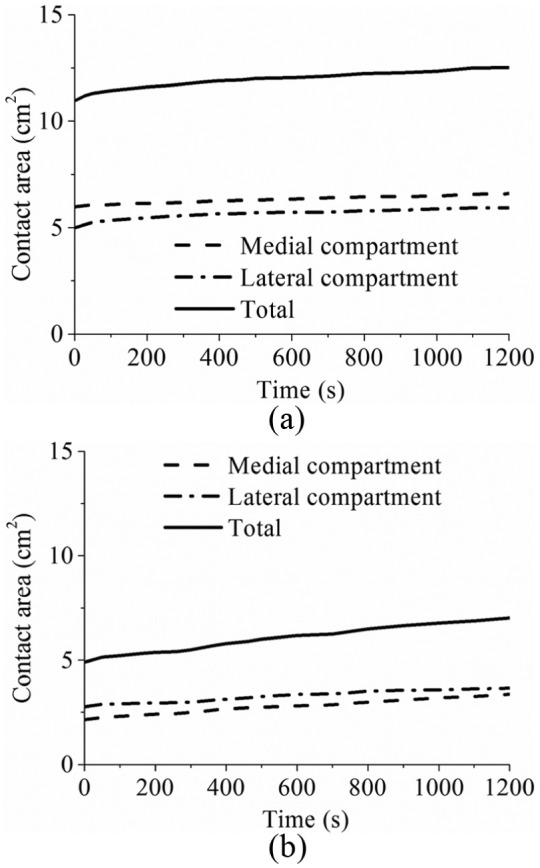
The contact area of (a) the intact and (b) the meniscectomy models within 1200 s of creep (the contact areas of each compartment and total contact area are shown).

The predicted time-dependent femoral vertical displacements for the intact and meniscectomy models are shown in Figure 3, and the corresponding contact areas are presented in Figure 4. Typical characteristics of the creep behaviour of hydrated soft tissues^29,54^ were found for the whole tibiofemoral joint: both the femoral vertical displacement and contact area increased with time. After 1200 s, the femoral vertical displacement of the intact model increased 74% (from 0.95 to 1.65 mm), while that of the meniscectomy model increased 128% (from 0.59 to 1.35 mm) (Figure 3). The total contact areas of the intact and meniscectomized joints were 10.98 and 4.92 cm^2^, respectively (Figure 4), when the load was just applied. They increased to 12.53 and 7.02 cm^2^, respectively, after 1200 s (Figure 4). The contact area of each separate compartment of the intact and meniscectomized joints also increased with time (Figure 4). During the whole creep period, the contact area of the medial compartment of the intact joint was larger than that of the lateral compartment (Figure 4(a)). However, the meniscectomized joint showed a different scenario: the contact area of the lateral compartment was larger (Figure 4(b)).

**Figure 5.**
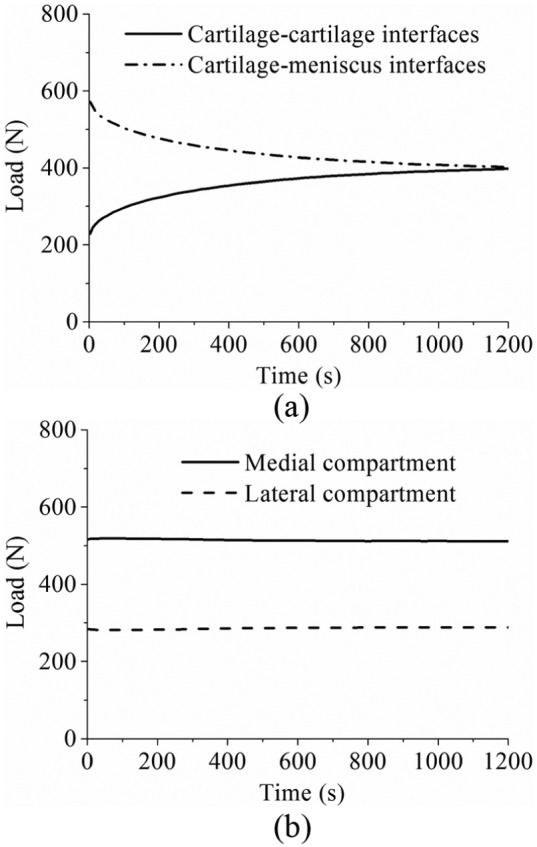
(a) The force transmitted by the cartilage–cartilage and cartilage–meniscus interfaces of the intact knee model during 1200 s of creep. (b) The load distribution at the medial and lateral compartments of the intact knee model within 1200 s of creep.

The time-dependent variation in the forces transmitted by the cartilage–cartilage and meniscus–cartilage interfaces of the intact joint is shown in Figure 5(a). When the load was just applied, 72% (572 N) was sustained by the meniscus–cartilage interface. As creep developed, more force was transferred to the cartilage–cartilage interface. At 1200 s, the load transmitted by the cartilage–cartilage and meniscus–cartilage interfaces was almost the same (Figure 5(a)). The variation in the load distributions between the lateral and medial compartments of the intact joint with time is shown in Figure 5(b). As expected,^55^ the medial compartment bore a larger proportion (65%) of load than the lateral compartment. Moreover, the load distribution between the lateral and medial compartments did not markedly vary with time (Figure 5(b)).

**Figure 6.**
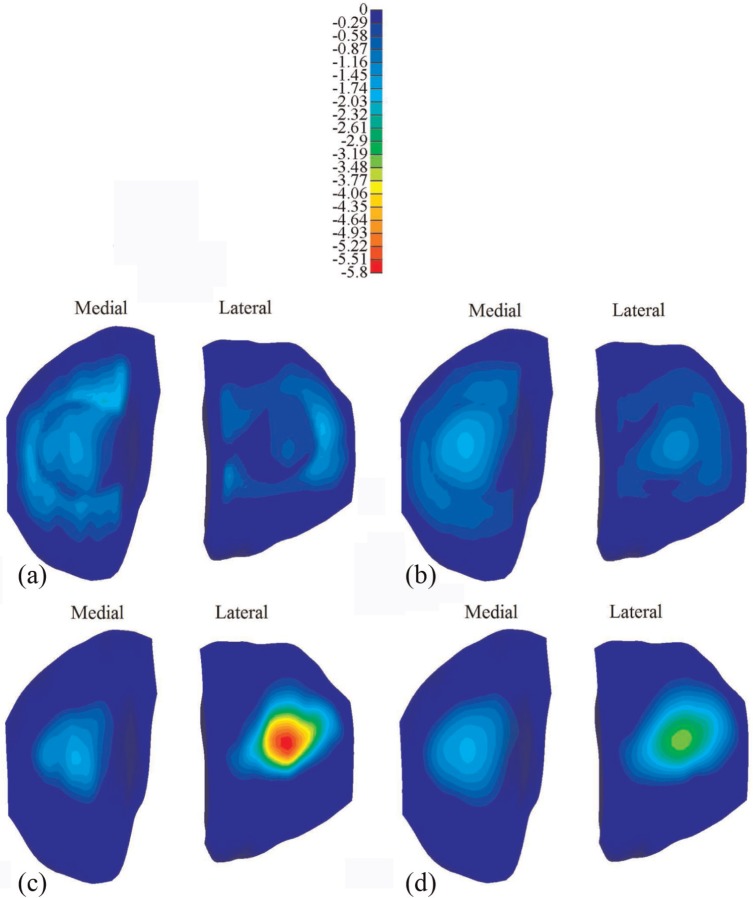
The maximum compressive stress (MPa) on the tibial cartilage: (a) the intact knee when the load was just applied, (b) the intact knee when the load was held for 1200 s, (c) the meniscectomy knee when the load was just applied and (d) the meniscectomy knee when the load was held for 1200 s.

The distribution of the maximum compressive stress at different instants is shown in Figure 6 for the intact and meniscectomized joints. During the whole creep period, the stress in the meniscectomized joint was considerably higher than that in the intact joint, with the peak values of the maximum compressive stress at 1 and 1200 s increased by 174% and 87% relative to intact values, respectively. The contact area of the cartilage–cartilage interfaces increased considerably with time, as shown by the increased light blue area in the cartilage–cartilage interfaces when compared between Figure 6(a) and (b). Moreover, there was a substantial reduction in stress in most regions of the cartilage–meniscus interfaces of the intact joint with increasing time (Figure 6(a) and (b)). Furthermore, the stress in the medial compartment of the intact joint was generally higher than that in the lateral side, whereas the stress in the lateral compartment of the meniscectomized joint was higher (Figure 6(c) and (d)) than that in the medial side.

**Figure 7.**
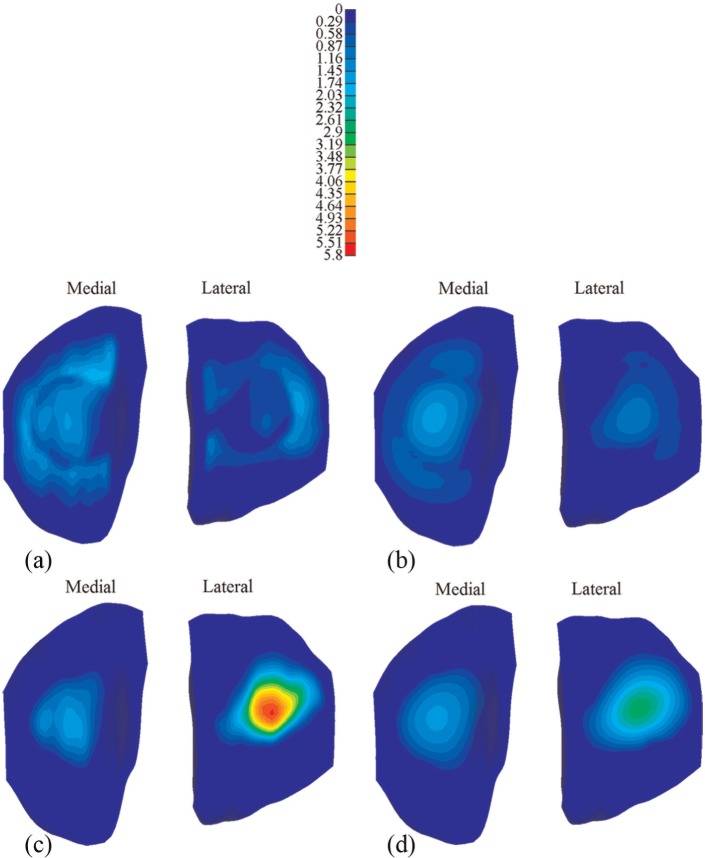
The fluid pressure (MPa) on the tibial cartilage: (a) the intact knee when the load was just applied, (b) the intact knee when the load was held for 1200 s, (c) the meniscectomy knee when the load was just applied and (d) the meniscectomy knee when the load was held for 1200 s.

**Figure 8.**
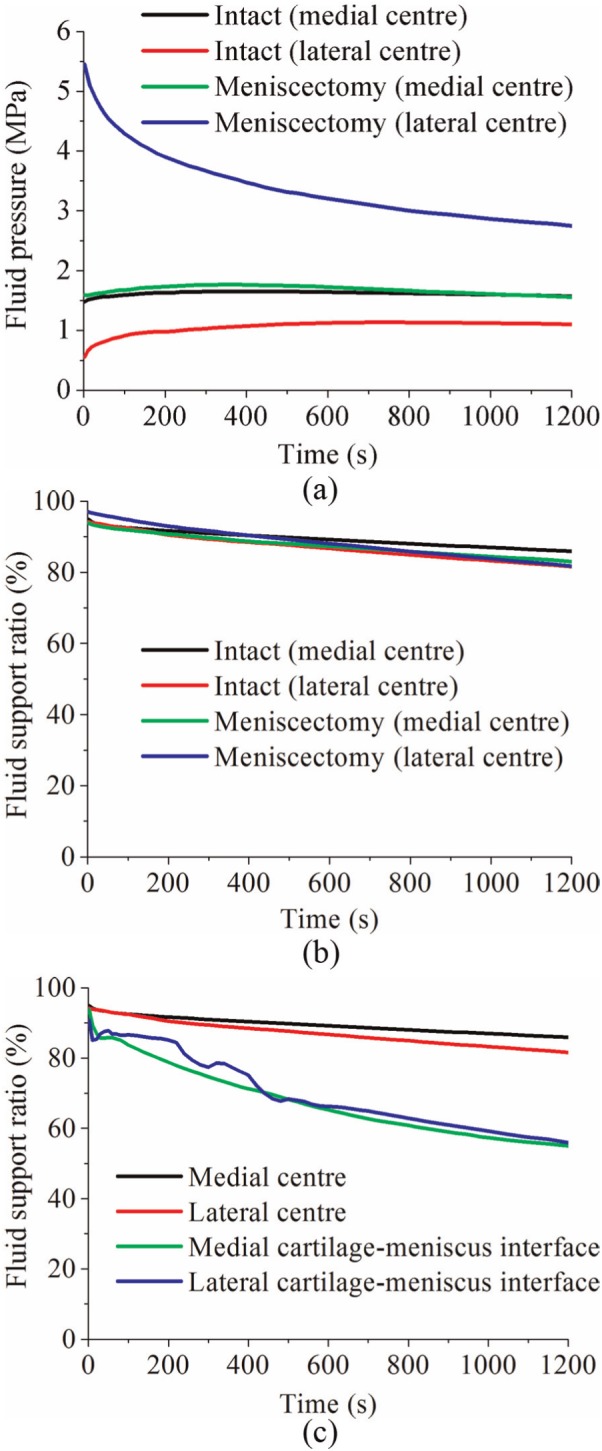
(a) The fluid pressure and (b) fluid support ratio at the condyle centres of the intact and meniscectomy models within 1200 s of creep, and (c) the comparison of fluid support ratio between different positions on the tibial cartilage of the intact joint within 1200 s of creep.

The corresponding comparison of the fluid pressure between the intact and meniscectomized joints is shown in Figure 7. The fluid pressure distributions in the intact and meniscectomized joints were consistent with the maximum compressive stress. The fluid pressure in the intact and meniscectomized joints remained remarkably high for 1200 s, especially at the compartment centres. At the medial compartment centre, the fluid pressure in both the intact and meniscectomized joints remained almost equal (Figure 8(a)). At the lateral compartment centre, the fluid pressure in the intact model remained constant with a slight increase during the first 200 s, while in the meniscectomy model, it reduced 50% after 1200 s (Figure 8(a)). However, when the fluid support ratio was compared, the differences between the four compartment centres were minor. The ratio was around 95% in all cases when the load was applied and remained as high as 80% at 1200 s (Figure 8(b)). Generally, the fluid support ratio at the cartilage–meniscus interfaces decreased considerably faster than the compartment centres (Figure 8(c)) because the cartilage–meniscus interfaces are close to the free-draining boundaries of the meniscus and cartilage.

## Discussion

Investigating the contact mechanics of the tibiofemoral joint using computational models is very challenging,^10^ since many of the important biomechanical aspects are difficult to implement. These aspects include the multiple contacts between the knee component tissues with complex geometries, the fibril-reinforced biphasic mechanical model, the finite deformation of the cartilage and menisci and the contact-dependent fluid flow boundary conditions. The time-dependent contact behaviour of the tibiofemoral joint under physiological loading and realistic fluid flow boundary conditions, which is important to learn the mechanical environment of the articular cartilage and meniscus within the whole joint, has not been fully understood. Therefore, the aim of this study was to develop a FE human tibiofemoral model considering the above conditions and to characterize the time-dependent contact behaviour of the joint under two-legged stance over extended periods.

Before the time-dependent contact behaviour was characterized, the validity of the model prediction was first assessed by comparison with data from the literature. The facts that the model outputs of instantaneous loads fell within the range found experimentally (Tables 2 and 3) and the time-dependent variation in the third principal strain in the compartment centres showed a similar trend to the in vivo measurement of the contact deformation (Figure 2) provide confidence that the model predictions were reasonable. There are, of course, limitations to this validation step. First, only femoral displacement and contact area were compared with instantaneous experiments because there are many restrictions on the measurements that can be practically taken in an experiment. In addition, there will be inevitable variations in the geometry and material properties of the experimental test specimens,^56–58^ while the model represents only one specific case. There were also some differences between the constraints applied in the experiments and this study. For example, Kurosawa et al.^49^ only allowed the axial translation between the femur and tibia. The anterior–posterior (AP) and medial–lateral (ML) translation and IE rotation between the femur and tibia were allowed by Morimoto et al.^50^ The differences in the experimentally measured displacements reported by Shrive et al.^52^ and Walker and Erkman^51^ reflect how variations in specimen and test set-up can affect the results, and the fact that this study predictions fall within these experimental values provides confidence that the model predictions are reasonable. Furthermore, to the authors’ knowledge, only one in vivo experimental study on the creep behaviour of the tibiofemoral joint under constant load has been published by Hosseini et al.^53^ Different from this study, one-legged stance was investigated by Hosseini et al., where the contact force applied to the joint can be estimated to be two times BW.^48^ Therefore, due to the lack of experimental data of a comparable functional activity, the time-dependent contact behaviour predicted in this study could not be directly validated. Further work is currently underway to develop an in vitro testing facility, and now that the computational methodology has been developed, it will be possible to generate specimen-specific models in the future to enable direct validation against corresponding experimental tests.

When the whole joint was subjected to a compressive load, the fluid pressurization played an important role in increasing the effective stiffness of the cartilage and meniscus. When the interstitial fluid flowed away from the loaded region with time, the effective stiffness of the cartilage and meniscus was thereby reduced. As a result, a larger contact area was required for the contact interfaces to balance the applied load, accompanied with the increased vertical displacement. Therefore, as expected, the contact area and femoral vertical displacement of the intact and meniscectomized joints increased with time (Figures 3 and 4). Due to the complex structure of the knee joint, little experimental work on the time-dependent contact behaviour of the knee joint has been published.^53,59,60^ In the only study that experimentally investigated the creep behaviour of the tibiofemoral joint,^53^ the cartilage–meniscus contact was not included because the motion and deformation of the meniscus are not detectable using the experimental techniques adopted by that study.^53^ Therefore, the time-dependent contact areas predicted in this study (Figure 4) are of considerable interest. Together with the predicted time-dependent femoral vertical displacement, they provide further understanding of the basic contact behaviour of the knee joint under the boundary conditions used.

Tibiofemoral load transmission has long been recognized as a key function of the meniscus.^61,62^ The human menisci are believed to transmit 30%–55% of the load in a standing position.^61,63^ The fraction of load transmitted by the menisci was also reported to be as high as 90% at the full extension position.^64^ In this study, when the load was just applied, the fraction transmitted by the menisci (72%, Figure 5) was consistent with the previous studies. This study also indicated that under creep conditions, the load transmitted by the menisci was dependent on time, whereby the applied load was gradually transferred to the cartilage–cartilage interfaces. These observations provide further insight into the mechanical environment of human knee joint and biomechanical function of the cartilage and menisci. This conclusion is different from that drawn in a previous study,^25^ in which it was thought that the menisci bore more load as creep developed. These differing conclusions may result from either the different geometry of the knee joint between this study and the previous study or the fact that the previous finding was derived from the increase in the first principal stress in the meniscus as creep developed.

Investigating how the load distributes in the two compartments of a tibiofemoral joint is important because this distribution is believed to relate to the development of OA in the medial and lateral compartments.^55^ This study showed that 65% of the load went through the medial compartment (Figure 5(b)). This result was consistent with the previous studies, in which it was reported that approximately 60%−70% of load may pass through the medial compartment.^55^ This study also showed that the load distribution between the medial and lateral compartments did not vary markedly with time under the studied loading conditions (Figure 5(b)). Such an understanding could be used to assess whether surgical interventions to limit OA progression are effective in altering this load distribution and in developing design criteria for tissue-engineered constructs. It should be noted that the medial offset of the loading position of this study played an important role in the load distribution between the medial and lateral compartments. If the load was applied at the joint centre, the load tended to equally distribute between the two compartments (see Appendix 2). The effect of shifting the loading position medially actually highlighted the importance of the adduction moment of the knee joint, which has been emphasized by previous studies.^23,65^

It should be noted that the double meniscectomy case studied in this study would be rarely performed today. This extreme case was chosen only to assess the functional behaviour of the cartilage in isolation, highlight the function of the menisci and demonstrate the sensitivity of the model to pathologic conditions.^37^ Moreover, varying degrees of meniscectomy may affect tibiofemoral alignment.^23,66–68^ Such a meniscectomy-induced change in joint alignment was not considered in the meniscectomy model (the same alignment as the intact joint was kept for the meniscectomized joint). This may be the reason why the contact area and compressive stress in the lateral compartment of the meniscectomy model were larger than those of the medial side although the loading position of the meniscectomy model was also medially moved. This result differs from the previous studies,^37,49^ in which the medial side of the double meniscectomized joint indicated larger contact area. Therefore, similar to other studies without considering the reposition of the femur and tibia, the results of the meniscectomy model in this study should be treated with caution.^68^ However, the comparison between the intact model and the meniscectomy model in this study did indicate the functions of the menisci. The menisci are believed to help increase contact area and reduce stress of the knee joint.^49,62,69^ Indeed, the reduction in the contact area (Figure 4) and increase in the compressive stress (Figure 6) after meniscectomy obtained in this study provide more evidence for this function of the menisci. This study also indicated that the menisci increased the contact area and decreased the stress in the cartilage for the whole creep period.

The prediction of the fluid pressure in the cartilage in this study may have important implications for cartilage degeneration. The fluid pressure protects the cartilage by shielding the solid phase from direct contact and excessive stress and deformation. Therefore, the high fluid support ratio at the compartment centres of the intact and meniscectomized joints (Figure 8(b)) may effectively protect the cartilage in these areas. The rapid decrease in the fluid pressure at the cartilage–meniscus interfaces (Figure 8(c)) may have adverse implications for the cartilage in these regions. This finding would agree with Qazi et al.^70^ where, based on homogeneity discrimination, the meniscus-covered region in the tibial cartilage appeared to be a site of early OA.

Although many important conclusions have been drawn from the FE knee models without considering the fluid pressure in the cartilage and menisci,^21,23,71^ these studies could not obtain the above insight related to the fluid pressure as well as other viscoelastic characteristics presented in this study. Compared with other studies that investigated the creep behaviour of the tibiofemoral joint,^25,26^ the loading value applied in this study was more physiological, and therefore, the conclusions may be more clinically relevant. Moreover, the inclusion of the contact-dependent fluid flow boundary conditions in this study enabled the prediction of the fluid pressure to be more theoretically valid.^15,19,20^

Due to the complexity of the time-dependent contact problem of the tibiofemoral joint, there are limitations in this study. First, the ligaments were not included to simplify the model.^27,28,34^ The ligaments stabilize the knee joint through restricting rotations and translations of femur with respect to tibia.^72^ The function of the ligaments was taken into account by the applied constraints in this study.^27^ For example, the AP translation between the tibia and femur was fully constrained^27,34^ to simulate the function of anterior cruciate ligament (ACL) and posterior cruciate ligament (PCL). However, such a constraint is a simplification of the physiological conditions because under the compressive load considered in this study, some AP and ML translation will occur if the ligaments are included.^73^ This simplification is likely to cause the predicted stress distribution to be translated in the transverse plane. However, the precise estimation of the effects of including the ligaments and the AP and ML translations requires a more elaborate model, which will be developed in the future.

Furthermore, the depth-dependent material inhomogeneity of cartilage, for example, the changes of the collagen fibre orientation, compressive modulus and permeability through the depth of the tissues, was not considered in this study. This inhomogeneity plays an important role in the mechanical behaviour of the cartilage of the knee joint,^27,74,75^ notably, enhancing the fluid support in the superficial zone.^74,76^ Therefore, the fluid pressure predicted in this study may be underestimated. The modelling methodology presented here could now be extended to investigate the effects of cartilage inhomogeneity through sensitivity studies and specimen-specific comparisons with experiments.

Despite the above limitations, this study provides further understanding of the mechanical environment of the human knee joint and the biomechanical functions of the cartilage and meniscus. The model developed in this study incorporated more realistic loading and fluid flow contact boundary conditions. Future work will use this model to examine the alteration in the time-dependent contact behaviour of the knee resulting from common clinical problems such as cartilage and meniscal defects^77–79^ and the performance of proposed repair techniques.^80–82^

## Figures and Tables

**Figure 9.**
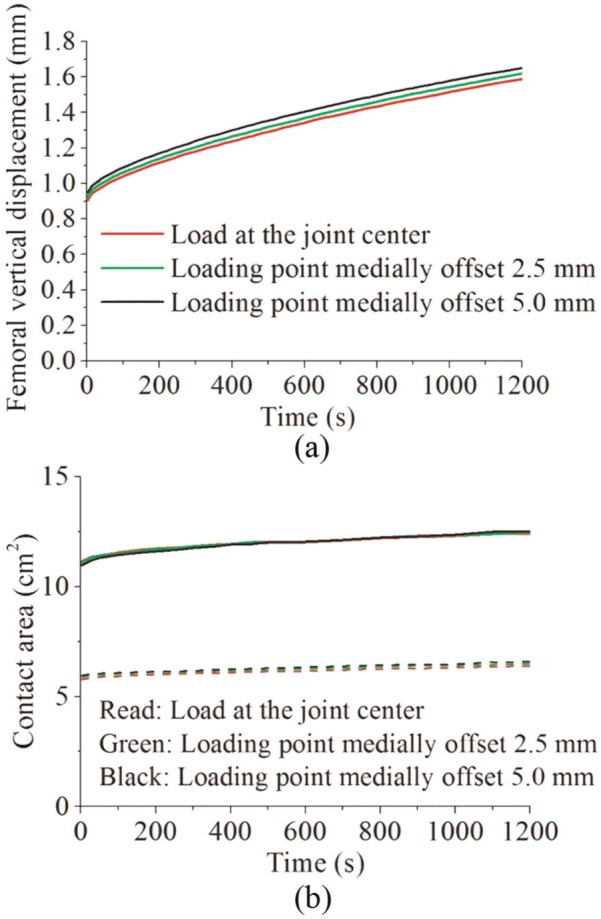
The effect of shifting the loading position on (a) the femoral vertical displacement (mm) and (b) the total contact area (solid lines) (cm^2^) and the medial contact area (dashed lines) (cm^2^) of the intact knee model.

**Figure 10.**
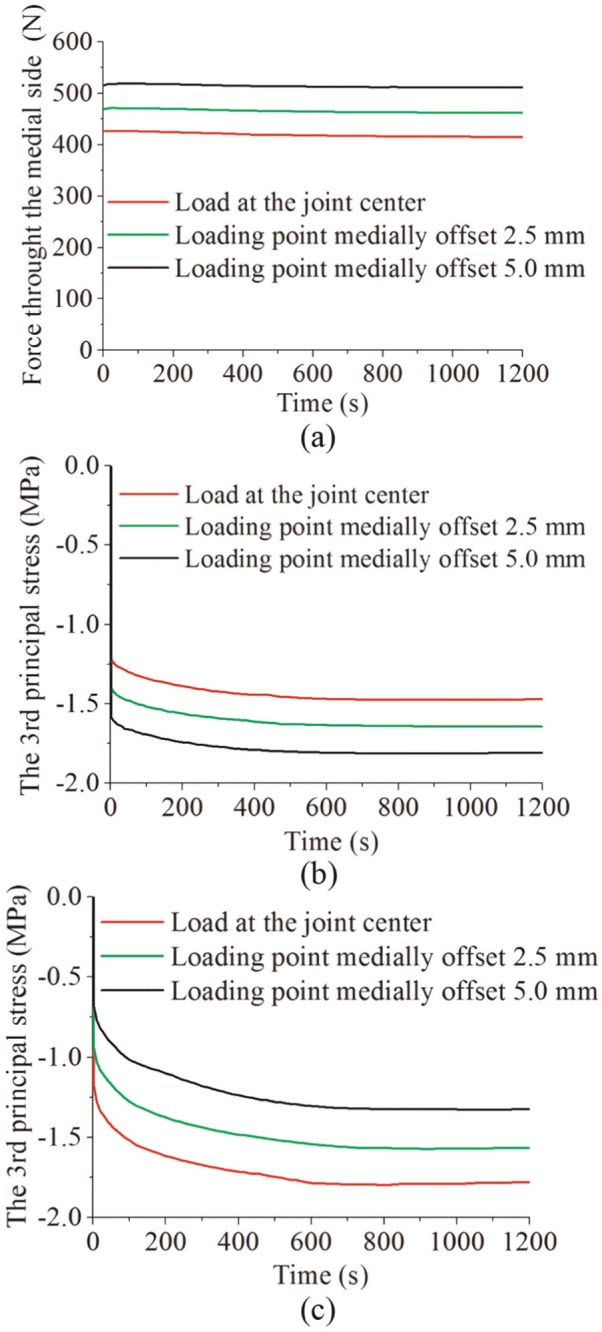
The effect of shifting the loading position on (a) the force (N) passing through the medial compartment, (b) the maximum compressive stress (MPa) at the contact centre of the medial compartment and (c) the maximum compressive stress (MPa) at the contact centre of the lateral compartment of the intact knee model.

## Appendix 1

### Governing equations for the biphasic fibril-reinforced material

The Cauchy stress tensor **σ** in a biphasic material represents the contributions of the interstitial fluid pressurization and solid matrix deformation:

(1) $$\text{σ}=-pI+{\text{σ}}^{e}$$

where *p* is the interstitial fluid pressure, **I** is the identity tensor and **σ**^*e*^ is the stress resulting from the solid matrix deformation (strain).

When a biphasic material is loaded, its interstitial fluid pressurizes. The fluid flows from regions of high pressure to regions of low pressure:

(2) w=−k·gradp

where **w** is the volumetric flux (flow rate per total area) of fluid relative to the solid matrix, grad *p* is the spatial gradient in the fluid pressure and **k** is the hydraulic permeability tensor representing the resistance to interstitial fluid flow within the porous solid.

The principal material behaviours that need to be characterized by constitutive relations are the dependence of stress in the solid matrix **σ**^*e*^ and hydraulic permeability **k** on solid matrix strain and porosity. If these constitutive relations are known, the interstitial fluid pressure *p* and solid matrix deformation (displacement vector) **u** are obtained by solving the balance of linear momentum and balance of mass equations for the biphasic mixture, subject to suitable boundary conditions. These balance equations are^83^

(3) divσ=0

(4) $$\text{div}\;(v^{s}+w)=0$$

where **v**^*s*^ is the solid matrix velocity, equal to the material time derivative of **u**. Note that equations (3) and (4) are partial differential equations with respect to *p* and **u** if equations (1) and (2) and the constitutive equations for **σ**^*e*^ and **k** are substituted into them.

In this study, the solid matrix of the biphasic materials was represented using a mixture of a non-fibrillar ground matrix of a neo-Hookean material and fibres. The compressive behaviour of the biphasic materials was represented by the neo-Hookean model while the tensile behaviour was dominated by the fibres. The total stress of the solid matrix, **σ**^*e*^, was then given by the sum of the fibre stress, **σ**_*f*_, and the ground matrix stress, **σ**_*m*_^84^

(5) $${\text{σ}}^{e}={\text{σ}}_{m}+{\text{σ}}_{f}$$

The ground matrix stress, **σ**_*m*_, was derived from the strain energy function of the neo-Hookean material, which is defined as^31,84^

(6) $$\Psi_{m}=\frac{\mu }{2}\left(I_{1}-3\right)-\mu \;\ln\;J+\frac{\lambda }{2}{\left(\ln\;J\right)}^{2}$$

where *I*_1_ and *I*_2_ are the first and second invariants of the right Cauchy–Green deformation tensor and *J* is the determinant of the deformation gradient tensor. *λ* and *μ* are the Lamé parameters, related to Young’s modulus *E* and Poisson’s ratio *ν* as follows

(7) $$\lambda =\frac{\nu E}{\left(1+\nu )(1-2\nu \right)},\;\mu =\frac{E}{2(1+\nu )}$$

Three orthogonal bundles of fibres were defined for each cartilage or meniscus component. For the cartilage, similar tensile properties were defined for the fibres in the three directions to represent a uniform distribution. The primary fibres of the menisci were oriented in the circumferential direction.^85^ A larger tensile modulus in the circumferential direction than in the radial direction was defined.^86^ The strain energy function for each bundle of fibre followed an exponential power law^84,87^

(8) $$\Psi_{f}=\frac{\xi }{\beta }{\left(I_{n}-1\right)}^{\beta }$$

where *β* is the power of exponential argument, *I_n_* is the square of the fibre stretch and *ξ* is the measure of the fibre tensile modulus. When *β* > 2, a smooth transition in the stress from compression to tension can be considered. The application of such a material model requires detailed tensile stress–strain characteristic curves for the menisci and cartilage. For the case of *β* = 2, the elasticity of the fibre at the strain origin (zero strain) reduces to 4*ξ*.^84^ Then, at the strain origin, the aggregate modulus in tension (*H*_+A_) of the cartilage or meniscus is *H*_A_ + 4*ξ*, where *H*_A_ is the aggregate modulus in compression. Therefore, when both *H*_A_ and *H*_+A_ are known, *ξ* can be determined. Therefore, due to the lack of the detailed tensile stress–strain characteristic curve of the cartilage and meniscus, *β* was assumed to be 2 in this study, allowing *ξ* for the cartilage and meniscus to be calculated from the equilibrium compressive and tensile modulus.

The dependence of the permeability of the cartilage and menisci on strain and direction^88,89^ was not considered here for simplicity, and it will be investigated in a future study. Therefore, the permeability of the tibial and femoral cartilage and menisci was simplified as a constant.

## Appendix 2

### The effect of the offset of the loading position on the contact behaviour of the intact knee

In order to investigate the effect of the medial offset of the loading position on the time-dependent contact behaviour of the intact knee joint, two more cases were also considered. In the first case, the vertical load of 800 N was applied at the joint centre, while the loading position was medially shifted 2.5 mm (equivalently, a vertical load of 800 N and an adduction moment of 2 N m applied at the joint centre) in the second case.

The comparison between the three cases showed that the offset of the loading position did not remarkably affect the femoral vertical displacement and the contact area. Compared with loading at the joint centre, medially shifting 5 mm caused a 5% increase in the femoral vertical displacement (Figure 9(a)). The change in the total contact area caused by the 5-mm medial offset was less than 1% (Figure 9(b)). The contact area that was shifted from the lateral side to the medial side by the 5-mm medial offset was only approximately 1.7% total contact area (Figure 9(b)).

The force distribution between the medial and lateral compartments and the stress in the two compartments were considerably affected by the offset of the loading position. If the load was applied at the joint centre, 53% of the load passed through the medial compartment (Figure 10(a)). The load passing through the medial compartment increased to 65% if the loading position was medially shifted 5 mm (Figure 10(a)). Since the increase in the contact area of the medial compartment caused by the 5-mm offset was only 1.7% (Figure 9(b)), the compressive stress in the medial condyle centre increased 22%, compared with loading at the joint centre (Figure 10(b)). Correspondingly, the compressive stress in the lateral condyle centre decreased 26% (Figure 10(c)).
